# Resistance to linezolid in *Staphylococcus aureus* by mutation, modification, and acquisition of genes

**DOI:** 10.1038/s41429-024-00778-4

**Published:** 2024-10-17

**Authors:** Wenjing Yang, Taoran Chen, Qi Zhou, Jiancheng Xu

**Affiliations:** 1 Center of Infectious Diseases and Pathogen Biology, The First Hospital of Jilin University, Changchun, China; 2 Department of Laboratory Medicine, The First Hospital of Jilin University, Changchun, China; 3 Department of Pediatrics, The First Hospital of Jilin University, Changchun, China

**Keywords:** Antimicrobial resistance, Clinical microbiology

## Abstract

Linezolid binds to the 50S subunit of the bacterial ribosome, inhibiting bacterial protein synthesis by preventing the formation of the initiation complex. Oxazolidinone antimicrobial drugs represent the last line of defense in treating *Staphylococcus aureus* infections; thus, resistance to linezolid in *S. aureus* warrants high priority. This article examines the major mechanisms of resistance to linezolid in *S. aureus*, which include: mutations in the domain V of 23S rRNA (primarily G2576); chromosomal mutations in the *rplC, rplD, and rplV* genes (encoding the ribosomal uL3, uL4, and uL22 proteins, respectively); the exogenous acquisition of the methylase encoded by the chloramphenicol-florfenicol resistance (*cfr*) gene; the endogenous methylation or demethylation of 23S rRNA; the acquisition of *optrA* and *poxtA* resistance genes; and the existence of the LmrS multidrug efflux pump. In conclusion, these mechanisms mediate resistance through mutations or modifications to the bacterial target, thereby reducing the affinity of linezolid for the peptidyl transferase center (PTC) binding site or by preventing the binding of linezolid to the PTC through a ribosomal protective effect. The existence of additional, unexplained resistance mechanisms requires further investigation and verification.

## Introduction

*S.aureus* is one of the leading causes of morbidity and mortality worldwide as an infectious agent, capable of causing a spectrum of infections ranging from moderately severe to fatal, including skin and soft-tissue infections, pneumonia, infective endocarditis, osteoarthritis infections, central nervous system infections, bloodstream infections, and toxic shock syndrome [1]. *S. aureus* infections were initially managed effectively with penicillin following its introduction in the 1940s, however, penicillin-resistant strains emerged rapidly [2]. Since then, *S. aureus* isolates have consistently demonstrated antibiotic resistance, with methicillin-resistant *Staphylococcus aureus* (MRSA) emerging as a particularly significant resistant strain [3].

Linezolid became the first oral oxazolidinone antibiotic to be approved by the U.S. Food and Drug Administration (FDA) for clinical applications in 2000. Since being introduced to the Chinese market in 2007, linezolid has shown potent antibacterial activity against the majority of Gram-positive bacteria and has become a key treatment option for MRSA [2]. Additionally, resistance to linezolid in *S. aureus* was reported within the first year of its clinical application in China [4, 5]. The 2022 findings from the China Antimicrobial Surveillance Network (CHINET) revealed that the resistance rate of methicillin-resistant coagulase-negative *Staphylococcus* (MRCNS) stood at 1.6%, and no linezolid-resistant MRSA cases were detected [6, 7]. According to the report detailing linezolid susceptibility testing results from 2015 for the LEADER Program, linezolid exhibited potent activity against *S. aureus*, inhibiting more than 99.9% of 3,031 isolates at ≤ 2 µg ml^−1^. Similarly, linezolid demonstrated coverage for 99.2% of coagulase-negative *staphylococci*, 99.7% of *enterococci*, and 100.0% of *Streptococcus pneumoniae*, the viridans group, and beta-hemolytic *streptococcus* isolates. Oxazolidinone antimicrobials remain the last resort for treating *S. aureus* infections, even though resistance to linezolid in *S. aureus* remains low. Consequently, it is imperative to carefully monitor resistance to linezolid in *S. aureus*, and clarifying the mechanism of this resistance is of urgent importance [8].

In this review, we discuss the potential pathways of *S. aureus* resistance, along with their prevalence and clinical significance, following linezolid’s authorization for clinical use over the past 23 years. This review aims to provide a theoretical foundation for the clinical selection of medications and for preventing the spread of resistant bacterial strains.

## The mechanism of action to linezolid

**Fig. 1:**
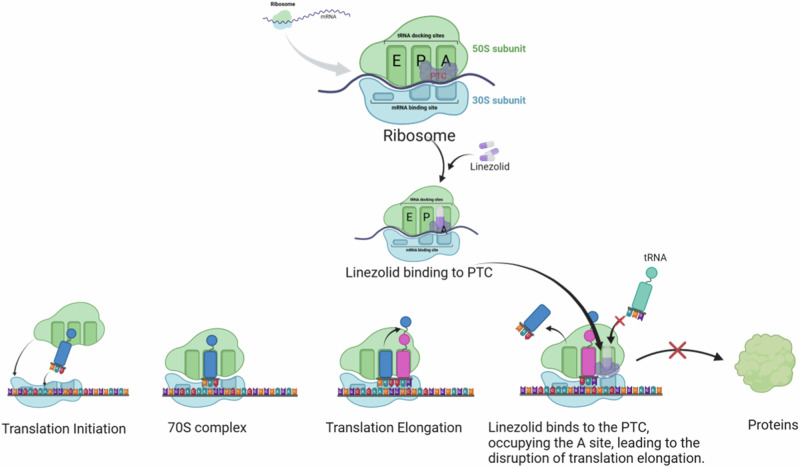
Linezolid can tightly bind to the PTC region of the ribosome, which is located near the A site. This binding allows linezolid to selectively regulate the binding of tRNA to the A site of the ribosome. Due to the overlap of linezolid with the position originally occupied by tRNA at the A site, when linezolid binds, it blocks the entry of tRNA, thus hindering protein synthesis spatially

Linezolid, as the first synthetic oxazolidinone antibacterial agent, possesses a unique chemical structure and mode of action [9]. Through in-depth analysis of the drug’s structure, we have found that oxazolidinones can bind with significant affinity and selectivity to the catalytic site on the 50S ribosomal subunit, which is specifically located at the ribosomal peptidyl transferase center, leading to a change in the positioning of tRNA [10]. Linezolid effectively inhibits bacterial protein synthesis by targeting the PTC, the active site on the large 50S ribosomal subunit. Single-molecule studies further confirm that linezolid can selectively regulate the binding of tRNA to the ribosomal A-site, rather than indiscriminately blocking the formation of peptide bonds. Upon binding to the PTC, linezolid creates a spatial obstruction at the A-site, thereby blocking protein synthesis and exerting its antimicrobial efficacy (Fig. 1). Linezolid demonstrates comprehensive antibacterial activity against a variety of Gram-positive bacteria, including strains resistant to other antibiotics, with a minimum inhibitory concentration (MIC) ranging from 0.5 to 4 µg ml^−1^ [5, 9–12].

## Mechanisms of resistance to linezolid

Although the inappropriate use of linezolid in clinical settings has contributed to the selection and spread of resistance in *Staphylococcus*, it is important to note that the resistance mechanisms themselves are not novel but rather represent the utilization of pre-existing genetic elements in response to the selective pressure exerted by antibiotic use [13]. Currently, the resistance situation is quite severe, and the main identified resistance mechanisms include the following six types: (1) mutations in the domain V of 23S rRNA; (2) mutations in the *rplC, rplD*, and *rplV* genes; (3) acquisition of plasmids carrying the *cfr* resistance gene through horizontal or vertical transmission; (4) methylation of the 23S rRNA to protect the ribosome from binding with linezolid; (5) ribosome protection by the OptrA and PoxtA proteins in the ABC-F family, preventing linezolid from binding to the ribosome; (6) increased efflux of linezolid by the LmrS multidrug efflux pump. The interaction of these complex mechanisms ultimately leads to the resistance of *S. aureus* to linezolid [14].

### Mutations in the domain V of 23S rRNA

Given that the domain V of 23S rRNA harbors the linezolid action site, any mutations or structural alterations within this region could influence bacterial resistance to linezolid [15]. The first clinical strain of linezolid-resistant *S. aureus*, identified in 2001, exhibited the G2576 mutation in the domain V of 23S rRNA, reducing the drug’s affinity for its target site [16]. Follow-up studies confirmed that mutations at this locus were predominant, occurring in 63.5% of linezolid-resistant *Staphylococcus aureus* (LRSA), 60.2% of linezolid-resistant coagulase-negative *staphylococci* (LRCoNS), and 57.5% of linezolid-resistant *enterococci* [14].

In a study investigating how oxazolidinone-class antibiotics interfere with the ribosomal peptidyl transferase center and affect tRNA positioning, it was revealed that G2576 is located at a position where it directly stacks onto one of the universally conserved residues, G2505, within the oxazolidinone binding site. In the crystals of the native *Deinococcus radiodurans* 50S ribosomal subunit (D50S)-oxazolidinone structure, the oxazolidine ring, a primary component of the drug, appears to stack against the base of U2504. Consequently, mutations at G2576 may confer resistance allosterically by altering the positioning of U2504. This is distinct from other mutation sites, which are primarily clustered around G2504 and its base-pairing partner C2452, indirectly affecting oxazolidinone binding by influencing the adjacent bases at these locations [17].

Additionally, research has established that *staphylococci* possess 5–6 copies of rRNA *(rrn)* operons, with resistance levels increasing alongside the number of mutant 23S rRNA alleles, while growth rates decrease due to mutation-mediated resistance in the 23S rRNA [18]. In an in vitro experiment, mutants exhibiting increased MIC of linezolid were generated by progressively exposing linezolid-sensitive cells to media containing escalating concentrations of the drug [19]. These mutants accumulated G2576 mutations across multiple copies of the 23S rRNA gene, a phenomenon that closely correlated with the degree of drug resistance [20, 21]. Locke et al. demonstrated, through serial passage of methicillin-susceptible *S. aureus* (MSSA) and MRSA in linezolid, that the MICs for MRSA and MSSA increased 32-fold and 64-fold, respectively, over 30 generations, indicating that mutations conferring linezolid resistance in the 23S rRNA can persist even without antibiotic pressure [22]. Most linezolid resistance mutations are centrally located between nucleotides 2400 and 2600 of the 23S rRNA [15, 23–26]. Additionally, specific point mutations in the 23S rRNA genes, including T2500, C2534, G2766, T2504, and G2447, have been identified as associated with resistance in clinical strains (Table 1) [22, 27, 28].

**Table 1:** Mutation sites on 23 s rRNA, L3, and L4 proteins were detected in linezolid-resistant Staphylococcus aureus

Type of mutation	Mutation site	Strains origin	Co-existence of other mutations or resistance genes	reference
Mutations in 23S rRNA	G2447	In vitro selection/clinical isolates	–	[45]
	U2500	In vitro selection/clinical isolates	–	[34]
	U2504	In vitro selection	–	[27]
	G2576	In vitro selection/clinical isolates	–	[27, 34, 45, 49]
	C2534	In vitro selection/clinical isolates	–	[45]
	T2571	In vitro selection	–	[50]
	A2572	In vitro selection	–	[50]
	G2234	Clinical isolates	–	[45, 51]
	G2621	Clinical isolates	–	[51]
	C2192	In vitro selection	–	[25]
	A2503	In vitro selection/clinical isolates	*cfr*	[27, 34]
	G2766	In vitro selection	–	[28]
Mutations in L3 protein	F127H146	In vitro selection	–	[37]
	G139R	Clinical isolates	G2576	[16]
	D145	Clinical isolates	–	[37]
	D145H146Y	Clinical isolates	*cfr*	[39]
	G152D	In vitro selection	G2447	[37]
	G152D	Clinical isolates	–	[52]
	G155R	In vitro selection	–	[37]
	G155R/M169L	In vitro selection	–	[37]
	M169G174	Clinical isolates	*cfr*	[39]
	G359U	In vitro selection	–	[53]
	T433-T435	Clinical isolates	–	[37]
Mutations in L4 protein	K68Q	In vitro selection	–	[37]
	G69A	Clinical isolates	G2576	[42]
	T70P	Clinical isolates	G2576	[42]

### Mutations in the *rplC*, *rplD*, and *rplV* genes

Linezolid targets the 50S large ribosomal subunit, specifically the binding sites closely associated with ribosomal proteins uL3, uL4, and uL22 [28]. Studies have shown that mutations in the *rplC*, *rplD*, and *rplV* genes on the chromosome, which encode these ribosomal proteins, alter the structure and stability of 23S rRNA [14], thereby affecting bacterial sensitivity to linezolid. However, resistance to linezolid in *S. aureus* caused by these mutations is relatively rare [29].

Although ribosomal protein uL3 is primarily located on the surface of the 50S subunit, it possesses two looped structures extending to the PTC. Since 2003, research has revealed that mutations in bacterial uL3 are associated with resistance to linezolid and other antibiotics. In this study, all detected uL3 mutations were concentrated in a central extension near the PTC. For instance, the G152D and G155R mutations are related to mycoplasma resistance [30–32]. The G152D mutation is located below a conserved mismatch site and may indirectly interfere with bases 2505 and 2506, reducing the affinity for oxazolidinones [33–36]. In *S. aureus*, mutations in the uL3 protein often occur simultaneously with the 23S rRNA G2576T mutation. Additionally, some studies have found that uL3 protein mutations (such as D145H146Y and M169G174) co-occur with the exogenously acquired cfr gene, which encodes an enzyme named chloramphenicol-florfenicol resistance protein, this enzyme can mediate resistance to linezolid [37].

In addition to the aforementioned mutations, other alterations in ribosomal protein uL3 are also associated with linezolid resistance in *Staphylococcus species*. When investigating the impact of G139D, L94V, D159, G152, T146Y and F84V mutations in uL3 on linezolid resistance, we observed that these changes structurally significantly affect the drug’s binding and mechanism of action. Through in silico analysis, we found that these mutations primarily influence the tertiary structure of uL3, particularly the α-helices and large coiled regions. Specifically, the mutations at D159 and G152 (transformed to Tyr and Asp, respectively) are located within a large coiled region. These amino acids, compared to the original ones, are larger and have different polarities. This alteration could lead to a significant deformation of the tertiary structure of uL3, thereby affecting its binding affinity with linezolid. Although the L94V substitution is also within a coiled region, it involves two amino acids of similar size and polarity, suggesting a smaller structural impact. Moreover, mutations such as T146Y and G139D may also influence the tertiary structure of uL3, thereby indirectly affecting its interaction with linezolid. These structural changes could result in alterations to the binding site of the drug to the ribosome or impact the ribosome’s function during synthesis, thereby affecting the sensitivity of bacteria to linezolid [38].

In summary, these mutations in uL3 protein may alter its structure, thereby influencing the interaction between linezolid and the ribosome, ultimately leading to linezolid resistance in bacteria. These findings provide important insights into the molecular mechanisms of linezolid resistance and offer a theoretical basis for the development of new strategies against drug resistance. However, these conclusions require experimental validation and further research to be fully substantiated.

The *rplD* gene encodes the uL4 protein located near the PTC, and mutations in this gene, including adjacent 6-bp deletions, may lead to a slight reduction in linezolid susceptibility. The extended tip of uL4 primarily interacts with 23S rRNA, stabilizing the key region of the PTC through charge neutralization and packaging interactions [39]. In *S.aureus*, the K68Q mutation in the uL4 protein can decrease the antibacterial effect. K68 interacts with the sugar-phosphate backbone of A2059 and G2061, collectively forming a pocket around A2503 [35]. The loss of compensatory charge due to the K68Q mutation can cause deformation of the pocket and subsequent movement of A2503 towards the PTC region, affecting the affinity for various types of antibiotics [35, 40]. Currently, clinical resistance to linezolid in *S.aureus* has been found in uL4 proteins containing G69A and T70P mutations [41].

Due to the proximity of the ribosomal protein uL22 gene to the linezolid binding site, mutations, deletions, or substitutions in uL22 amino acids may affect the spatial structure of the PTC, thereby being associated with linezolid resistance [35, 41, 42].

### The methylation of 23S rRNA by the Cfr methyltransferase encoded by the *cfr* gene

In the current arsenal of antibiotics for treating infectious diseases in humans, numerous drugs target the bacterial ribosome and exert their efficacy by inhibiting protein synthesis. Some of these drugs bind to the PTC region and function by competing with aminoacyl-transfer RNA (aa-tRNA) substrates at the PTC or its vicinity. However, there are also ribosome-targeting antibiotics that act on multiple other functional sites on the ribosome, such as the decoding center and the GTPase-associated center, among others [43, 44]. However, the natural evolutionary response of pathogenic bacteria to antibiotic treatment involves the acquisition and spread of resistance genes that are already present in microbial communities. Among these genes, the *cfr* gene is particularly common in *S. aureus* resistance to linezolid. Recent studies have identified new variants of the *cfr* gene, such as *cfr(B)*, *cfr(C), cfr(D)*, and *cfr(E)* [45–47].

The *cfr* methyltransferase encoded by the *cfr* gene confers resistance to a broad range of PTC-targeting antibiotics by performing C8-methylation on the universally conserved adenosine residue A2503 of the 23S ribosomal RNA [48, 49]. This modification is located at the center of the ribosome, near the A site of the PTC, affecting the binding of various antibiotics, including phenicols, lincosamides, oxazolidinones, pleuromutilins and streptogramins A (collectively known as PhLOPSA), as well as hygromycin A and 16-membered macrolides (16MMs) [50, 51]. The *cfr* gene was first identified in 2000 in *Mammaliicoccus sciuri* (formerly *Staphylococcus sciuri*) and has since been detected in a wide array of Gram-positive and Gram-negative bacteria [52].

The mechanism of *cfr*-mediated resistance involves two primary components: first, the direct steric hindrance of antibiotics by the m8A2503 group, and second, the *cfr*-induced rearrangement of nucleotide A2062, leading to an allosteric rearrangement of the drug-binding pocket. These mechanisms act in concert, preventing a variety of chemically unrelated antibiotic classes from binding to *cfr*-modified ribosomes. Structural studies indicate that the m8A2503 group spatially overlaps with the binding sites of PTC-targeting drugs, supporting the “direct spatial conflict” model. However, the degree of spatial overlap between the C8-methyl group and certain PTC-binding drugs is relatively small, suggesting the presence of additional resistance mechanisms [53].

In particular, nucleotide A2062 in *cfr-*modified ribosomes is unable to rotate, which prevents additional binding interactions with antibiotics and thereby promotes resistance through an allosteric mechanism. This mechanism plays a significant role in resistance to phenicols and oxazolidinone antibiotics. Compared to the A2062 mutation, the replacement of A2062 with a pyrimidine (U or C) results in strong resistance to most A site-targeting antibiotics, further underscoring the importance of A2062 in drug-ribosome binding [53].

In summary, structural information about *cfr*-modified ribosomes and their complexes with antibiotics provides an important starting point for the development of next-generation drugs against multidrug-resistant pathogens. Designing antibiotics that can form new interactions with the A site of *cfr*-methylated ribosomes may be an effective strategy to combat pathogens expressing the *cfr* gene.

### 23S rRNA modification protects ribosomes from binding to linezolid

*Spr0333* is an hypothetical gene in *Streptococcus pneumoniae* that encodes a 385-amino acid protein containing a S-adenosylmethionine (SAM)-dependent methyltransferase (PFAM UPF0020) domain. The Spr0333 protein shares 32% sequence identity with a domain of the *E. coli* gene product RlmL, which encodes an rRNA methyltransferase responsible for the N2-methylation of G2445 in the 23S rRNA [54]. Mutations in Spr0333 are located within its conserved SAM methyltransferase domain, including non-synonymous mutations and frameshift deletions, which may lead to the inactivation of Spr0333 and are associated with linezolid resistance [55].

Methylation of ribosomal RNA is a common mechanism of acquired resistance to antibiotics such as erythromycin [55]. Endogenous RNA methylases may play a role in protecting 23S rRNA from natural xenobiotics [45]. Primer extension assays demonstrate that Spr0333 exhibits 23S rRNA methylation activity, and susceptibility testing confirms that different mutations in Spr0333 are directly linked to linezolid resistance [55].

Orthologs of Spr0333 are found in many bacterial species, including *S.aureus*. In a previous study of vancomycin intermediate MRSA (VISA) from the NARSA collection (www.narsa.net) [46], we confirmed that three isolates, NRS 119, 127, and 271, were resistant to linezolid. The ortholog of Spr0333 in *S. aureus* is SAV1444 (named according to the nomenclature of *S. aureus* Mu50) [46], which shares about 45% identity with the *Streptococcus pneumoniae* ortholog. In the isolate NRS119, which had the highest MIC against linezolid, the sequence of the SAV1444 gene revealed a 39-bp deletion in the SAM-methylation domain, No mutations were observed in the two other VISA isolates that were less resistant to linezolid. NRS271 and NRS127 were selected in vitro for increased linezolid resistance, and their SAV1444 gene was resequenced. A 60-bp deletion was observed in the in vitro linezolid-selected NRS271, indicating that the Spr0333 ortholog SAV1444 is directly related to the linezolid resistance of this *S. aureus* strain [55].

### ABC-F protein through a ribosomal protection to prevent linezolid from binding to ribosomes

The ATP-binding cassette (ABC) protein superfamily is a class of proteins widely distributed across all life domains, primarily involved in the energy-dependent transport of molecules across biological membranes [47]. This superfamily comprises multiple subfamilies, the majority of which are composed of typical ABC transporters with two ABC domains and two transmembrane domains (TMDs) [56]. However, members of the ABC-E and ABC-F subfamilies are neither fused with TMDs nor genetically associated with TMDs in operons [56, 57]. The ABC-F subfamily is abundant in both eukaryotes and bacteria, and these members have been implicated in a variety of biological processes, including DNA repair [58, 59], translational control [60], and resistance to antibiotics targeting bacterial protein synthesis [57].

The classification of ABC-F proteins primarily includes the following types: (1) Antibiotic Resistance Expressers (AREs): These ABC-F proteins are closely related to the antibiotic resistance of bacteria, capable of protecting bacteria from the attack of specific antibiotics [60]. The expression of these proteins is typically inducible, meaning they are only activated when bacteria encounter specific antibiotics. For example, OptrA and PoxtA belong to this category of proteins. (2) House-keeping ABCFs: These ABC-F proteins are essential for maintaining the normal function of bacterial ribosomes and translational control. They play a fundamental role in the growth and metabolism of bacteria and are not directly involved in antibiotic resistance. For instance, EttA (Efflux Transporter) in *Escherichia coli* is a house-keeping protein [61]. (3) Ribosome Protection Proteins (RPPs): These ABC-F proteins can bind to ribosomes and protect them from certain antibiotics that typically target the bacterial protein synthesis mechanism [62]. (4) Translation regulatory proteins [63], and so on.

In pathogens, the interaction or binding of ABC-F ARE proteins with the 50S subunit is not always observed. This interaction is typically conditional, occurring when the pathogen is exposed to specific exogenous compounds (e.g., antibiotics) [60]. These antibiotics target the 50S ribosomal subunit of bacteria, thereby inhibiting protein synthesis. When antibiotics are absent, ABC-F ARE proteins may not bind to the 50S subunit, or their binding may not impact the normal function of bacteria. However, upon exposure to these antibiotics, the activity of ABC-F ARE proteins may be induced or enhanced, leading to their binding to the 50S subunit to prevent antibiotic binding or action, thus conferring antibiotic resistance. Therefore, the binding of ABC-F ARE proteins to the 50S subunit is a responsive mechanism aimed at protecting bacteria from antibiotics. This binding is dynamic and depends on whether the pathogen is exposed to specific antimicrobial drugs [64].

In the ABC-F ARE subfamily, OptrA and PoxtA proteins are two significant members that play a crucial role in conferring resistance to linezolid in *S.aureus* [65]. The genes that encode these proteins are located on mobile genetic elements (MGEs), which can facilitate their transfer between different bacterial strains. This family of proteins provides resistance by directly protecting the target from the effects of antibiotics, rather than through drug efflux. *OptrA* gene is responsible for plasmid-mediated linezolid resistance, first identified in a human-derived Enterococcus faecium isolate and also found in MRSA, commonly co-existing with the *cfr* gene. The resistance mediated by OptrA primarily manifests as tolerance to oxazolidinone and phenylacetamide antibioticss [66, 67]. The catalytic domain of OptrA contains two glutamate (E) motifs that exhibit ATP hydrolysis activity, providing preliminary evidence for the resistance associated with OptrA [68]. The structure of the OptrA protein includes two nucleotide-binding domains (NBD1 and NBD2), which are connected by a spiral connector and do not contain known transmembrane domains [69]. These catalytic glutamate residues are located at the ATP-binding sites of the two NBD domains and are crucial for ATP hydrolysis.

The mechanism of linezolid resistance mediated by OptrA involves several key steps. OptrA binds to the 50S subunit and disrupts its structure, preventing antibiotics from binding to the ribosome. This mechanism directly affects the structure and function of the ribosome, particularly the alteration of the peptidyl transferase center, which is the site where antibiotics bind to inhibit protein synthesis. This structure allows OptrA to affect ribosomal function, especially at the drug-binding site. This mechanism differs from the traditional drug efflux mechanism, which involves ATP hydrolysis to drive antibiotics out of the bacterial cytoplasm [70].

The clinical significance of OptrA-mediated resistance lies in its ability to confer resistance to a range of important antibiotic classes, including oxazolidinones, lincomycins, macrolides, oxazolidinones, phenylacetamides, prulifloxacins, and streptogramin A and B groups [40, 71]. This resistance is prevalent in pathogens and has been observed in some clinical isolates mediated by the *optrA* gene against oxazolidinones and phenylacetamides. Therefore, the OptrA-mediated resistance mechanism is a complex biochemical process involving direct interactions between proteins and ribosomes, providing a scientific foundation for the development of new antimicrobial resistance strategies.

PoxtA is a resistance determinant belonging to the ABC-F ARE proteins [66]. It was initially detected in the genome of a linezolid-resistant MRSA strain isolated from a cystic fibrosis patient. The *poxtA* gene encodes a protein with 32% identity to the OptrA protein and possesses structural features for mediating antibiotic resistance through ribosome protection. The resistance mechanism of PoxtA protein involves perturbing the P-site tRNA, causing it to move approximately 4 Å towards the ribosome, equivalent to a shift of one amino acid during translation. This perturbation may interfere with the drug binding site by altering the conformation of the nascent peptide chain attached to the ribosome [72]. The structural studies provide crucial insights into how PoxtA mediates linezolid resistance. The specific mechanism of action of PoxtA involves the ATP-dependent regulation of the peptidyl transferase center, altering its stereochemical properties and global conformation, thereby influencing the binding geometry of the P-site tRNA. This mechanism enables PoxtA to protect bacteria from antibiotics targeting the ribosome.The *poxtA* gene has been shown to functionally contribute to the reduction of resistance to at least three classes of antiribosomal antibiotics, including phenylacetamides, oxazolidinones, and tetracyclines [66].

These two genes emergence and spread may gradually increase the level of resistance to linezolid and other drugs, posing challenges to clinical treatment. Therefore, monitoring the prevalence of these resistance genes in clinical and veterinary isolates and studying their interaction mechanisms of the encoded proteins with ribosomes are crucial for understanding and addressing the resistance of *S.aureus* to linezolid.

### LmrS efflux pump facilitates linezolid resistance in *S. aureus*

A study characterized the LmrS efflux pump from *S. aureus*, a member of the major facilitator superfamily, as a secondary active multidrug efflux pump with 14 predicted transmembrane domains. The expression of the *lmrS* gene cloned into a multicopy plasmid within the host organism conferred resistance to various antimicrobials. Among the antibacterial agents tested, the MIC for the oxazolidinone drug linezolid increased 16-fold [73, 74]. In conclusion, LmrS represents a critical determinant of antimicrobial resistance in *S. aureus*. Further research into the distribution of this gene among clinical isolates and the levels of LmrS expression is required to elucidate its precise role in the physiology and pathogenesis of *S. aureus*, particularly its contribution to antimicrobial resistance [74].

## Summary

**Fig. 2:**
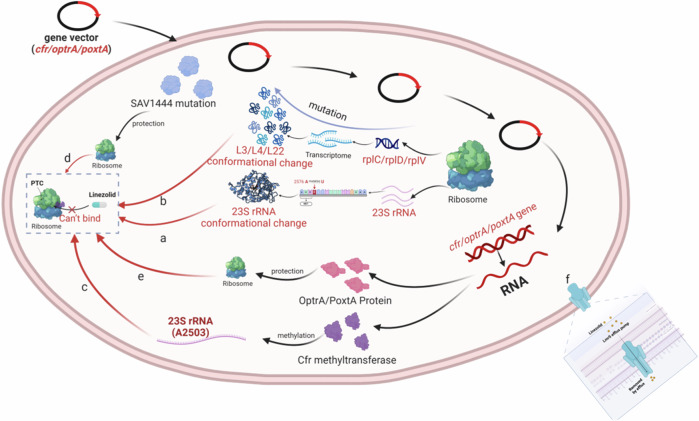
**a** The domain V of 23S rRNA is where the site of action of linezolid is located, and any site mutation and structural change of the point mutation in this region results in a decrease in the affinity of the drug for the target site leading to bacterial resistance. **b** The *rplC, rplD*, and *rplV* genes encode ribosomal proteins that are in close proximity to the linezolid binding site in the ribosome, and mutations in these genes, which are concentrated in the central region adjacent to the PTC, lead to a decrease in the susceptibility of *S. aureus* to linezolids by affecting 23S rRNA structure and stability. **c** The product encoded by the *cfr* gene is an rRNA methylase that affects drug binding to bacterial ribosomes by adding a methyl group to the C-8 position of the adenine residue (A2503) at position 2503 of the bacterial ribosomal 23S rRNA, thereby affecting the binding of the drug to the bacterial ribosome leading to resistance. **d** The SAV144 protein is an endogenous methyltransferase that catalyzes the methylation of 23S rRNA G2445, which when inactivated by mutations removes its methylation function and results in bacterial resistance to linezolid. **e** The ABC-F family of proteins encoded by the *optrA* and *poxtA* genes confer antibiotic resistance by interacting with ribosomes and displacing bound drugs, thereby conferring antibiotic resistance through a ribosomal protective mechanism. **f**: *S. aureus* possesses the LmrS protein, localized on a multicopy plasmid, which confers resistance to a wide range of antibiotics and is capable of increasing the MIC of the oxazolidinone drug linezolid 16-fold

Fig. 2 is a summary. Firstly, the G2576 mutation in the domain V of 23S rRNA occurred in 63.5% of LRSA cases, with a positive correlation between the number of cumulative mutations and the degree of resistance, highlighting G2576 as a prevalent and significant mechanism of resistance in *S. aureus*. Secondly, resistance to linezolid, mediated by mutations in the ribosomal proteins uL3, uL4, and uL22, predominantly involves the uL3 protein. The cumulative mutations in the *rplC* gene, encoding uL3, correlate positively with linezolid’s MIC. Mutations in ribosomal proteins often coexist with mutations in the domain V of 23S rRNA or the *cfr* gene, both mediating linezolid resistance in *S. aureus*. Thirdly, the *cfr* gene, primarily located on plasmids and often coexisting with removable progenitors, has a broad distribution and rapid spread, significantly reducing antimicrobial drug efficacy. Its prevention and control are critical. Additionally, endogenous methylation or demethylation alterations of 23S rRNA and LmrS efflux pumps rarely cause resistance to linezolid in *S. aureus*. Finally, the *optrA* and *poxtA* genes, carried on mobile genetic elements, can be transmitted across genetically distinct lineages without antimicrobial selection pressure. Recent international surveillance indicates that linezolid resistance remains rare; however, the widespread dissemination of *optrA* across all continents suggests it will become the primary resistance mechanism, warranting significant attention. In summary, point mutations or the acquisition of a single resistance gene seldom result in high-level bacterial resistance; instead, resistant isolates typically exhibit multiple resistance genes and mutations.

## Conclusion

Mechanisms of resistance to linezolid in *S. aureus* primarily encompass: (1) mutations in domain V of the 23S rRNA; (2) mutations in the r*plC, rplD*, and *rplV* genes; (3) methylation of the 23S rRNA gene by the protein encoded by the *cfr* gene.; (4) alterations in the modification of 23S rRNA; (5) ribosomal protection by ABC-F proteins; (6) the presence of the LmrS multidrug efflux pump. These resistance mechanisms operate either by mutations or modifications that reduce linezolid’s affinity to the PTC binding site or by ribosome protection effects that prevent linezolid’s binding to the PTC. Among these, the most prevalent and significant resistance mechanisms are mutations in the 23S rRNA within the ribosomal PTC region and the acquisition of *cfr* resistance genes. The trend of linezolid resistance in *S. aureus* warrants continuous monitoring, with the existence of additional, unexplained resistance mechanisms, both clinical and laboratory-derived, requiring further investigation and verification.

## References

[1] Cheung GYC, Bae JS, Otto M. Pathogenicity and virulence of Staphylococcus aureus. Virulence. 2021;12:547–69. 33522395 10.1080/21505594.2021.1878688 PMC7872022

[2] Ying F, Yonglu H, Jiachang C, Xiaowei L, Rong Z, Hongyan L. Antibiotic resistance profile and mechanism of linezolid-resistant Staphylococcus aureus strains. Chin J Infect Chemother. 2016;16:477–80.

[3] Turner NA, Sharma-Kuinkel BK, Maskarinec SA, Eichenberger EM, Shah PP, Carugati M, et al. Methicillin-resistant Staphylococcus aureus: an overview of basic and clinical research. Nat Rev Microbiol. 2019;17:203–18. 30737488 10.1038/s41579-018-0147-4 PMC6939889

[4] Shariati A, Dadashi M, Chegini Z, van Belkum A, Mirzaii M, Khoramrooz SS, et al. The global prevalence of Daptomycin, Tigecycline, Quinupristin/Dalfopristin, and Linezolid-resistant Staphylococcus aureus and coagulase-negative staphylococci strains: a systematic review and meta-analysis. Antimicrob Resist Infect Control. 2020;9:56. 32321574 10.1186/s13756-020-00714-9 PMC7178749

[5] Han X, Zou G, Liu J, Yang C, Du X, Chen G, et al. Mechanisms of linezolid resistance in Staphylococcus capitis with the novel mutation C2128T in the 23S rRNA gene in China. BMC Microbiol. 2022;22:203. 35987607 10.1186/s12866-022-02616-9 PMC9392311

[6] CHINET Releases 2022 China Bacterial Drug Resistance Surveillance Results in China. 2022 [cited 2023 0015/1/1]Available from: https://www.iivd.net/article-31711-1.html

[7] Shuo G, Wanging Z, Hong Z, Hui Z, Yan Z, Zhifeng Z, et al. Analysis on the resistance mechanism of linezolid-resistant Staphylococcus aureus. Chin J Clin Lab Sci. 2020;38:613–6.

[8] Pfaller MA, Mendes RE, Streit JM, Hogan PA, Flamm RK. Five-year summary of in vitro activity and resistance mechanisms of linezolid against clinically important gram-positive cocci in the United States from the LEADER Surveillance Program (2011 to 2015). Antimicrob Agents Chemother. 2017;61:e00609–17. 28483950 10.1128/AAC.00609-17 PMC5487612

[9] Belousoff MJ, Eyal Z, Radjainia M, Ahmed T, Bamert RS, Matzov D, et al. Structural basis for linezolid binding site rearrangement in the staphylococcus aureus ribosome. mBio. 2017;8:e00395–17. 28487427 10.1128/mBio.00395-17 PMC5424203

[10] Vazquez-Laslop N, Mankin AS. Context-specific action of ribosomal antibiotics. Annu Rev Microbiol. 2018;72:185–207. 29906204 10.1146/annurev-micro-090817-062329 PMC8742604

[11] Esposito S, Blasi F, Curtis N, Kaplan S, Lazzarotto T, Meschiari M, et al. New Antibiotics for Staphylococcus aureus Infection: An Update from the World Association of Infectious Diseases and Immunological Disorders (WAidid) and the Italian Society of Anti-Infective Therapy (SITA). Antibiotics. 2023;12:742. 37107104 10.3390/antibiotics12040742 PMC10135047

[12] Diekema DJ, Jones RN. Oxazolidinone antibiotics. Lancet. 2001;358:1975–82. 11747939 10.1016/S0140-6736(01)06964-1

[13] Ikeda-Dantsuji Y, Hanaki H, Nakae T, Takesue Y, Tomono K, Honda J, et al. Emergence of linezolid-resistant mutants in a susceptible-cell population of methicillin-resistant Staphylococcus aureus. Antimicrob Agents Chemother. 2011;55:2466–8. 21357291 10.1128/AAC.01548-10 PMC3088184

[14] Yueru T, Min L. The situation of Staphylococci resistance to Linezolid and the mechanisms. Chin J Clin Laboratory Manag. 2014, (01)(2):16–20.

[15] Turner AM, Lee J, Gorrie CL, Howden BP, Carter GP. Genomic insights into last-line antimicrobial resistance in multidrug-resistant staphylococcus and vancomycin-resistant enterococcus. Front Microbiol. 2021;12:637656. 33796088 10.3389/fmicb.2021.637656 PMC8007764

[16] Khodabux R, Mariappan S, Sekar U. Detection of a Novel G2603T Mutation in cfr Harboring Linezolid-Resistant Staphylococcus haemolyticus : first report from India. J Lab Phys. 2023;15:207–11. 10.1055/s-0042-1757419 PMC10264111 37323596

[17] Wilson DN, Schluenzen F, Harms JM, Starosta AL, Connell SR, Fucini P. The oxazolidinone antibiotics perturb the ribosomal peptidyl-transferase center and effect tRNA positioning. Proc Natl Acad Sci USA. 2008;105:13339–44. 18757750 10.1073/pnas.0804276105 PMC2533191

[18] Tsiodras S, Gold HS, Sakoulas G, Eliopoulos GM, Wennersten C, Venkataraman L, et al. Linezolid resistance in a clinical isolate of Staphylococcus aureus. Lancet. 2001;358:207–8. 11476839 10.1016/S0140-6736(01)05410-1

[19] Meka VG, Gold HS. Antimicrobial resistance to linezolid. Clin Infect Dis. 2004;39:1010–5. 15472854 10.1086/423841

[20] Besier S, Ludwig A, Zander J, Brade V, Wichelhaus TA. Linezolid resistance in Staphylococcus aureus: gene dosage effect, stability, fitness costs, and cross-resistances. Antimicrob Agents Chemother. 2008;52:1570–2. 18212098 10.1128/AAC.01098-07 PMC2292563

[21] Suzuki K, Saito M, Hanaki H. Increased copy number of 23S ribosomal RNA gene with point mutation in MRSA associated with linezolid resistance in a patient treated with long-term linezolid. J Infect Chemother. 2023;29:481–4. 36736701 10.1016/j.jiac.2023.01.019

[22] Stefani S, Bongiorno D, Mongelli G, Campanile F. Linezolid resistance in staphylococci. Pharm. 2010;3:1988–2006. 10.3390/ph3071988 PMC4036669 27713338

[23] Sujuan L. A study of linezolid resistant mechanisms and their molecular basis in Staphylococcus aureus. PhD, Zhejiang University, 2015.

[24] Howe RA, Wootton M, Noel AR, Bowker KE, Walsh TR, Macgowan AP. Activity of AZD2563, a novel oxazolidinone, against Staphylococcus aureus strains with reduced susceptibility to vancomycin or linezolid. Antimicrob Agents Chemother. 2003;47:3651–2. 14576139 10.1128/AAC.47.11.3651-3652.2003 PMC253812

[25] Livermore DM, Warner M, Mushtaq S, North S, Woodford N. In vitro activity of the oxazolidinone RWJ-416457 against linezolid-resistant and -susceptible staphylococci and enterococci. Antimicrob Agents Chemother. 2007;51:1112–4. 17210773 10.1128/AAC.01347-06 PMC1803128

[26] Livermore DM, Mushtaq S, Warner M, Woodford N. Activity of oxazolidinone TR-700 against linezolid-susceptible and -resistant staphylococci and enterococci. J Antimicrob Chemother. 2009;63:713–5. 19164418 10.1093/jac/dkp002

[27] Bonilla H, Huband MD, Seidel J, Schmidt H, Lescoe M, Mccurdy SP, et al. Multicity outbreak of linezolid-resistant Staphylococcus epidermidis associated with clonal spread of a cfr-containing strain. Clin Infect Dis. 2010;51:796–800. 20726771 10.1086/656281

[28] Xiuli X, Shan Z, Jiayun L, Xiaoke H, Peihong Y, Yueyun M. An analytical study of 23S rRNA point mutation-mediated clinical infection with linezolid-resistant MRSA. 4th SICCMAC. Qingdao, Shandong, China; 2014.

[29] Tewhey R, Gu B, Kelesidis T, Charlton C, Bobenchik A, Hindler J, et al. Mechanisms of linezolid resistance among coagulase-negative staphylococci determined by whole-genome sequencing. mBio. 2014;5:e814–e894. 10.1128/mBio.00894-14 PMC4030478 24915435

[30] Gentry DR, Rittenhouse SF, Mccloskey L, Holmes DJ. Stepwise exposure of Staphylococcus aureus to pleuromutilins is associated with stepwise acquisition of mutations in rplC and minimally affects susceptibility to retapamulin. Antimicrob Agents Chemother. 2007;51:2048–52. 17404009 10.1128/AAC.01066-06 PMC1891380

[31] Kosowska-Shick K, Clark C, Credito K, Mcghee P, Dewasse B, Bogdanovich T, et al. Single- and multistep resistance selection studies on the activity of retapamulin compared to other agents against Staphylococcus aureus and Streptococcus pyogenes. Antimicrob Agents Chemother. 2006;50:765–9. 16436741 10.1128/AAC.50.2.765-769.2006 PMC1366917

[32] Miller K, Dunsmore CJ, Fishwick CW, Chopra I. Linezolid and tiamulin cross-resistance in Staphylococcus aureus mediated by point mutations in the peptidyl transferase center. Antimicrob Agents Chemother. 2008;52:1737–42. 18180348 10.1128/AAC.01015-07 PMC2346621

[33] Long KS, Vester B. Resistance to linezolid caused by modifications at its binding site on the ribosome. Antimicrob Agents Chemother. 2012;56:603–12. 22143525 10.1128/AAC.05702-11 PMC3264260

[34] Locke JB, Hilgers M, Shaw KJ. Mutations in ribosomal protein L3 are associated with oxazolidinone resistance in staphylococci of clinical origin. Antimicrob Agents Chemother. 2009;53:5275–8. 19805557 10.1128/AAC.01032-09 PMC2786331

[35] Locke JB, Hilgers M, Shaw KJ. Novel ribosomal mutations in Staphylococcus aureus strains identified through selection with the oxazolidinones linezolid and torezolid (TR-700). Antimicrob Agents Chemother. 2009;53:5265–74. 19752277 10.1128/AAC.00871-09 PMC2786364

[36] Cui L, Wang Y, Li Y, He T, Schwarz S, Ding Y, et al. Cfr-mediated linezolid-resistance among methicillin-resistant coagulase-negative staphylococci from infections of humans. PLoS ONE. 2013;8:e57096. 23437319 10.1371/journal.pone.0057096 PMC3577776

[37] Locke JB, Morales G, Hilgers M, GC K, Rahawi S, Jose PJ, et al. Elevated linezolid resistance in clinical cfr-positive Staphylococcus aureus isolates is associated with co-occurring mutations in ribosomal protein L3. Antimicrob Agents Chemother. 2010;54:5352–5. 20837755 10.1128/AAC.00714-10 PMC2981277

[38] Ikonomidis A, Grapsa A, Pavlioglou C, Demiri A, Batarli A, Panopoulou M. Accumulation of multiple mutations in linezolid-resistant Staphylococcus epidermidis causing bloodstream infections; in silico analysis of L3 amino acid substitutions that might confer high-level linezolid resistance. J Chemother. 2016;28:465–8. 27077930 10.1080/1120009X.2015.1119373

[39] Klein DJ, Moore PB, Steitz TA. The roles of ribosomal proteins in the structure assembly, and evolution of the large ribosomal subunit. J Mol Biol. 2004;340:141–77. 15184028 10.1016/j.jmb.2004.03.076

[40] Kehrenberg C, Schwarz S, Jacobsen L, Hansen LH, Vester B. A new mechanism for chloramphenicol, florfenicol and clindamycin resistance: methylation of 23S ribosomal RNA at A2503. Mol Microbiol. 2005;57:1064–73. 16091044 10.1111/j.1365-2958.2005.04754.x

[41] Roman F, Roldan C, Trincado P, Ballesteros C, Carazo C, Vindel A. Detection of linezolid-resistant Staphylococcus aureus with 23S rRNA and novel L4 riboprotein mutations in a cystic fibrosis patient in Spain. Antimicrob Agents Chemother. 2013;57:2428–9. 23459489 10.1128/AAC.00208-13 PMC3632889

[42] Mendes RE, Deshpande LM, Farrell DJ, Spanu T, Fadda G, Jones RN. Assessment of linezolid resistance mechanisms among Staphylococcus epidermidis causing bacteraemia in Rome, Italy. J Antimicrob Chemother. 2010;65:2329–35. 20841419 10.1093/jac/dkq331

[43] Shen J, Wang Y, Schwarz S. Presence and dissemination of the multiresistance gene cfr in Gram-positive and Gram-negative bacteria. J Antimicrob Chemother. 2013;68:1697–706. 23543608 10.1093/jac/dkt092

[44] Vester B. The cfr and cfr-like multiple resistance genes. Res Microbiol. 2018;169:61–66. 29378339 10.1016/j.resmic.2017.12.003

[45] Toh SM, Mankin AS. An indigenous posttranscriptional modification in the ribosomal peptidyl transferase center confers resistance to an array of protein synthesis inhibitors. J Mol Biol. 2008;380:593–7. 18554609 10.1016/j.jmb.2008.05.027 PMC5367387

[46] Drummelsmith J, Winstall E, Bergeron MG, Poirier GG, Ouellette M. Comparative proteomics analyses reveal a potential biomarker for the detection of vancomycin-intermediate Staphylococcus aureus strains. J Proteome Res. 2007;6:4690–702. 17997515 10.1021/pr070521m

[47] Davidson AL, Dassa E, Orelle C, Chen J. Structure, function, and evolution of bacterial ATP-binding cassette systems. Microbiol Mol Biol Rev. 2008;72:317–64. 18535149 10.1128/MMBR.00031-07 PMC2415747

[48] Giessing AM, Jensen SS, Rasmussen A, Hansen LH, Gondela A, Long K, et al. Identification of 8-methyladenosine as the modification catalyzed by the radical SAM methyltransferase cfr that confers antibiotic resistance in bacteria. RNA. 2009;15:327–36. 19144912 10.1261/rna.1371409 PMC2648713

[49] Long KS, Poehlsgaard J, Kehrenberg C, Schwarz S, Vester B. The cfr rRNA methyltransferase confers resistance to phenicols, lincosamides, oxazolidinones, pleuromutilins, and streptogramin a antibiotics. Antimicrob Agents Chemother. 2006;50:2500–5. 16801432 10.1128/AAC.00131-06 PMC1489768

[50] Smith LK, Mankin AS. Transcriptional and translational control of the mlr operon, which confers resistance to seven classes of protein synthesis inhibitors. Antimicrob Agents Chemother. 2008;52:1703–12. 18299405 10.1128/AAC.01583-07 PMC2346656

[51] Polikanov YS, Starosta AL, Juette MF, Altman RB, Terry DS, Lu W, et al. Distinct tRNA accommodation intermediates observed on the ribosome with the antibiotics hygromycin A and A201A. Mol Cell. 2015;58:832–44. 26028538 10.1016/j.molcel.2015.04.014 PMC4458074

[52] Madhaiyan M, Wirth JS, Saravanan VS. Phylogenomic analyses of the Staphylococcaceae family suggest the reclassification of five species within the genus Staphylococcus as heterotypic synonyms, the promotion of five subspecies to novel species, the taxonomic reassignment of five Staphylococcus species to Mammaliicoccus gen. nov., and the formal assignment of Nosocomiicoccus to the family Staphylococcaceae. Int J Syst Evol Microbiol. 2020;70:5926–36. 33052802 10.1099/ijsem.0.004498

[53] Aleksandrova EV, Wu K, Tresco B, Syroegin EA, Killeavy EE, Balasanyants SM, et al. Structural basis of cfr-mediated antimicrobial resistance and mechanisms to evade it. Nat Chem Biol. 2024;20:867–76. 38238495 10.1038/s41589-023-01525-w PMC11325235

[54] Lesnyak DV, Sergiev PV, Bogdanov AA, Dontsova OA. Identification of Escherichia coli m2G methyltransferases: I. the ycbY gene encodes a methyltransferase specific for G2445 of the 23 S rRNA. J Mol Biol. 2006;364:20–25. 17010378 10.1016/j.jmb.2006.09.009

[55] Feng J, Lupien A, Gingras H, Wasserscheid J, Dewar K, Legare D, et al. Genome sequencing of linezolid-resistant Streptococcus pneumoniae mutants reveals novel mechanisms of resistance. Genome Res. 2009;19:1214–23. 19351617 10.1101/gr.089342.108 PMC2704432

[56] Dean M, Rzhetsky A, Allikmets R. The human ATP-binding cassette (ABC) transporter superfamily. Genome Res. 2001;11:1156–66. 11435397 10.1101/gr.184901

[57] Kerr ID. Sequence analysis of twin ATP binding cassette proteins involved in translational control, antibiotic resistance, and ribonuclease L inhibition. Biochem Biophys Res Commun. 2004;315:166–73. 15013441 10.1016/j.bbrc.2004.01.044

[58] Murat D, Bance P, Callebaut I, Dassa E. ATP hydrolysis is essential for the function of the Uup ATP-binding cassette ATPase in precise excision of transposons. J Biol Chem. 2006;281:6850–9. 16407313 10.1074/jbc.M509926200

[59] Burgos ZM, Alessandri K, Murat D, El AC, Dassa E. C-terminal domain of the Uup ATP-binding cassette ATPase is an essential folding domain that binds to DNA. Biochim Biophys Acta. 2010;1804:755–61. 19948254 10.1016/j.bbapap.2009.11.017

[60] Sharkey L, O’Neill AJ. Antibiotic resistance ABC-F proteins: bringing target protection into the limelight. ACS Infect Dis. 2018;4:239–46. 29376318 10.1021/acsinfecdis.7b00251

[61] Ousalem F, Ngo S, Oiffer T, Omairi-Nasser A, Hamon M, Monlezun L, et al. Global regulation via modulation of ribosome pausing by the ABC-F protein EttA. Nat Commun. 2024;15:6314. 39060293 10.1038/s41467-024-50627-z PMC11282234

[62] Ero R, Kumar V, Su W, Gao YG. Ribosome protection by ABC-F proteins-Molecular mechanism and potential drug design. Protein Sci. 2019;28:684–93. 30746819 10.1002/pro.3589 PMC6423996

[63] Ero R, Yan XF, Gao YG. Ribosome protection proteins-“new” players in the global arms race with antibiotic-resistant pathogens. Int J Mol Sci. 2021;22:5356. 34069640 10.3390/ijms22105356 PMC8161019

[64] Wilson DN, Hauryliuk V, Atkinson GC, O’Neill AJ. Target protection as a key antibiotic resistance mechanism. Nat Rev Microbiol. 2020;18:637–48. 32587401 10.1038/s41579-020-0386-z

[65] Singh KV, Weinstock GM, Murray BE. An Enterococcus faecalis ABC homologue (Lsa) is required for the resistance of this species to clindamycin and quinupristin-dalfopristin. Antimicrob Agents Chemother. 2002;46:1845–50. 12019099 10.1128/AAC.46.6.1845-1850.2002 PMC127256

[66] Antonelli A, D’Andrea MM, Brenciani A, Galeotti CL, Morroni G, Pollini S, et al. Characterization of poxtA, a novel phenicol-oxazolidinone-tetracycline resistance gene from an MRSA of clinical origin. J Antimicrob Chemother. 2018;73:1763–9. 29635422 10.1093/jac/dky088

[67] Pang S, Boan P, Lee T, Gangatharan S, Tan SJ, Daley D, et al. Linezolid-resistant ST872 Enteroccocus faecium harbouring optrA and cfr (D) oxazolidinone resistance genes. Int J Antimicrob Agents. 2020;55:105831. 31669743 10.1016/j.ijantimicag.2019.10.012

[68] Xiao-Bo Z, Lin W, Tie-Dong W, Ling Z, Da-Cheng W. Confirmation of two key active sites of the new drug resistance protein OptrA. Chin J Vet Sci. 2017;37:717–20.

[69] Murina V, Kasari M, Takada H, Hinnu M, Saha CK, Grimshaw JW, et al. ABCF ATPases Involved in protein synthesis, ribosome assembly and antibiotic resistance: structural and functional diversification across the tree of life. J Mol Biol. 2019;431:3568–90. 30597160 10.1016/j.jmb.2018.12.013 PMC6723617

[70] Su W, Kumar V, Ding Y, Ero R, Serra A, Lee B, et al. Ribosome protection by antibiotic resistance ATP-binding cassette protein. Proc Natl Acad Sci USA. 2018;115:5157–62. 29712846 10.1073/pnas.1803313115 PMC5960329

[71] Schwarz S, Werckenthin C, Kehrenberg C. Identification of a plasmid-borne chloramphenicol-florfenicol resistance gene in Staphylococcus sciuri. Antimicrob Agents Chemother. 2000;44:2530–3. 10952608 10.1128/aac.44.9.2530-2533.2000 PMC90098

[72] Ousalem F, Singh S, Chesneau O, Hunt JF, Boel G. ABC-F proteins in mRNA translation and antibiotic resistance. Res Microbiol. 2019;170:435–47. 31563533 10.1016/j.resmic.2019.09.005

[73] Koeth LM, Difranco-Fisher JM, Mccurdy S. A reference broth microdilution method for dalbavancin in vitro susceptibility testing of bacteria that grow aerobically. J Vis Exp 2015;103:53028. 10.3791/53028 PMC4692595 26381422

[74] Floyd JL, Smith KP, Kumar SH, Floyd JT, Varela MF. LmrS is a multidrug efflux pump of the major facilitator superfamily from Staphylococcus aureus. Antimicrob Agents Chemother. 2010;54:5406–12. 20855745 10.1128/AAC.00580-10 PMC2981259

